# Scaling correction in pediatric center-of-pressure analysis: a dual-track computational framework for standardized balance assessment

**DOI:** 10.3389/fbioe.2026.1857252

**Published:** 2026-07-13

**Authors:** Liwa Sha, Tzu-Cheng Lin, Wen Hsin Chiu

**Affiliations:** 1 Department of Sports Training, Jilin Sport University, Changchun, Jilin, China; 2 Department of Education and Learning Technology, National Tsing Hua University, Hsinchu, Taiwan; 3 Department of Kinesiology, National Tsing Hua University, Hsinchu, Taiwan

**Keywords:** anthropometric normalization, center of pressure, computational validation, dual-track analysis, pediatric postural control, scaling correction, stabilometry

## Abstract

**Objective:**

In this study, we developed and validated a standardized computational framework for pediatric balance assessment by integrating anthropometric normalization and a dual-track analytical architecture into a unified center-of-pressure (COP) signal-processing pipeline.

**Methods:**

This study included 90 typically developing children. They were stratified into age groups of 4, 6, and 10 years (n = 30 per group), balanced by sex. The children performed bipedal stance tasks under eyes-open and eyes-closed conditions. Static balance was assessed using a Zebris force platform (120 Hz). COP sway area, mediolateral displacement, and anteroposterior displacement were measured. Two parallel datasets were processed that included raw COP signals and leg length–normalized COP signals. Both datasets were subjected to 2 (sex) × 3 (age) analysis of variance to evaluate scaling sensitivity. Coefficients of variation were computed to assess system-level dispersion.

**Results:**

Anthropometric normalization markedly increased parameter sensitivity. Under the eyes-open condition, normalized COP sway area and anteroposterior displacement revealed age effects not detectable in the raw dataset, indicating scaling bias in uncorrected COP signals. The dual-track comparison confirmed that normalization functions as a computational validation component rather than a statistical add-on. For most COP parameters, the coefficients of variation exceeded 50%, indicating substantial interindividual variability consistent with developmental heterogeneity rather than measurement error.

**Conclusion:**

The proposed framework advances pediatric balance assessment from a descriptive developmental comparison to a standardized, bias-aware signal-processing architecture. By integrating anthropometric scaling correction and dual-track validation into a unified COP signal-processing pipeline, the framework enhances scaling sensitivity, inferential validity, and reproducibility. It is readily deployable within intelligent clinical screening systems and supports the development of age-stratified reference databases and early detection algorithms for pediatric neuromotor assessment.

## 1 Introduction

Assessment of static postural stability by using center-of-pressure (COP) metrics is common in biomechanics and rehabilitation engineering. COP parameters derived from force or pressure platforms are widely used to quantify postural sway characteristics, such as sway amplitude, directional displacement, and variability; this approach represents a standard method for objective evaluation of postural control (Winter, 1995; Ruhe et al., 2010). Differences in body dimensions may influence COP-derived stabilometric measures and complicate comparisons among children of different ages. Anthropometric characteristics have been shown to affect postural sway measurements, potentially introducing systematic bias when balance performance is compared across individuals with different body sizes (Chiari et al., 2020). From a biomechanical perspective, comparisons among individuals of different body sizes require consideration of scaling effects because body dimensions can systematically influence movement-related variables. Hof (1996) proposed that dimensionless normalization procedures can improve comparability across individuals by accounting for anthropometric differences. In standing balance tasks, leg length may be particularly relevant because it approximates the effective pendulum length of the body during postural control. During early childhood, rapid increases in height, leg length, and body mass alter the mechanical properties of the inverted pendulum model governing upright stance. Consequently, raw COP displacement signals reflect both neuromotor control and mechanical scaling components related to body geometry. This mixed composition reduces measurement invariance across age groups and thus cross-sectional comparability.

Although normalization techniques have been widely applied in gait and biomechanical analyses to improve dimensionless comparability (Hof, 1996; Mobbs et al., 2025), these approaches are typically implemented as isolated preprocessing procedures. In contrast, the present study does not propose a new scaling equation. Rather, it embeds anthropometric normalization within a dual-track computational validation architecture that enables simultaneous evaluation of raw and normalized COP signals. This framework allows scaling sensitivity, inferential robustness, and potential anthropometric bias to be systematically quantified within a single analytical pipeline. Standardization has been identified as an important methodological consideration in stabilometric assessment because measurement outcomes may be influenced by multiple non-physiological factors (Maiorov et al., 2025). However, the potential influence of anthropometric normalization on developmental inference in pediatric populations remains insufficiently investigated. Consequently, COP-based developmental studies often rely on raw parameters without validating whether statistical differences reflect true neuromotor maturation or anthropometric bias. This methodological limitation represents not only a statistical concern but also an engineering deficiency in signal-processing design. Without scaling correction, age-related comparisons risk overestimation of developmental effects. Furthermore, clinically meaningful application of stabilometric assessment requires reproducible and standardized processing algorithms. Engineering robust measurement systems requires the evaluation of parameter sensitivity across processing conditions. Divergent outcomes between normalized and raw datasets indicate scaling bias in signals. Therefore, assessing the effect of normalization should be considered a system validation procedure rather than a form of descriptive statistical comparison.

To address the lack of a standardized signal-processing framework in pediatric stabilometry, we developed and validated a computational normalization architecture for COP analysis in children. A leg length–based anthropometric scaling correction model was used to minimize geometric interference in COP signals. Leg-length normalization may improve the interpretability of COP measures by reducing the influence of body-size-related variation. Among the available anthropometric scaling variables, leg length was selected because it represents the effective pendulum length of the lower-limb system during quiet standing and is directly associated with the mechanical characteristics of COP displacement. Other anthropometric variables, including stature, body mass, and estimated center-of-mass height, may also influence balance performance; however, their relationship with COP kinematics is generally less direct. Accordingly, the present study focuses on leg-length normalization as a biomechanically grounded proof-of-concept implementation of the proposed framework. This mode was embedded within a dual-track analytical pipeline in which raw and normalized datasets are processed in parallel under a reproducible statistical framework. The primary objectives in implementing this mode are to assess age- and sex-related differences in static balance performance and to determine whether anthropometric normalization influences inferential outcomes, thereby detecting potential scaling bias in developmental COP measurements. Age-related differences in pediatric balance are often assessed through descriptive comparisons of postural performance metrics.

The primary innovation of the present study is not the normalization procedure itself, which follows established biomechanical scaling principles (Hof, 1996), but the integration of normalization into a dual-track computational validation framework. Therefore, evaluating COP variables using both raw and leg-length-normalized data may provide complementary information regarding developmental changes in pediatric balance performance. In addition, potential age and sex interactions were explored because developmental changes in postural control may not necessarily occur uniformly across sexes. This architecture enables direct comparison between raw and normalized outputs and allows the influence of anthropometric scaling on developmental inference to be quantified systematically. By integrating scaling correction, dual-track processing, and variability metrics into a unified architecture, this study advances pediatric COP analysis from observational reporting to engineering-based measurement validation and reproducible quantitative assessment.

## 2 Subjects and methods

### 2.1 Study cohort

This study included 90 typically developing children. They were stratified into three age groups: 4, 6, and 10 years (n = 30 per group). The participant, rather than individual COP samples, served as the statistical unit of analysis throughout the study. Each group had an equal sex distribution (15 boys and 15 girls). Anthropometric characteristics, such as height, body mass, and leg length, were recorded before balance assessment to support subsequent scaling correction (Table 1). This study excluded children with a ≤6-month-old history of musculoskeletal or neurological disorders affecting gait or postural control, those requiring assistive walking devices, and those using medications known to influence central nervous system function.

**TABLE 1:** Characteristics of the study cohort.

Group	Sex	Age (y)	Mass (kg)	Height (cm)	Leg length (cm)
4 (30)	M (15)	4.67 ± 0.71	15.98 ± 1.19	102.87 ± 4.01	50.3 ± 3.86
	F (15)	4.75 ± 0.85	15.63 ± 3.29	101.0 7 ± 6.09	49.94 ± 3.53
6 (30)	M (15)	6.88 ± 0.62	23.3 8 ± 4.61	119.22 ± 3.61	60.13 ± 2.57
	F (15)	6.88 ± 0.59	19.51 ± 2.58	114.08 ± 8.21	57.48 ± 4.94
10 (30)	M (15)	10.43 ± 0.79	36.51 ± 8.69	141.57 ± 10.72	74.26 ± 6.31
	F (15)	10.47 ± 0.81	32.13 ± 8.03	141.3 ± 10.70	75.41 ± 7.27

The study protocol was approved by the Institutional Review Board of National Tsing Hua University (approval number: 11302HT023). Written informed consent was obtained from the participants’ parents or legal guardians before their participation.

### 2.2 Data collection

**FIGURE 1:**
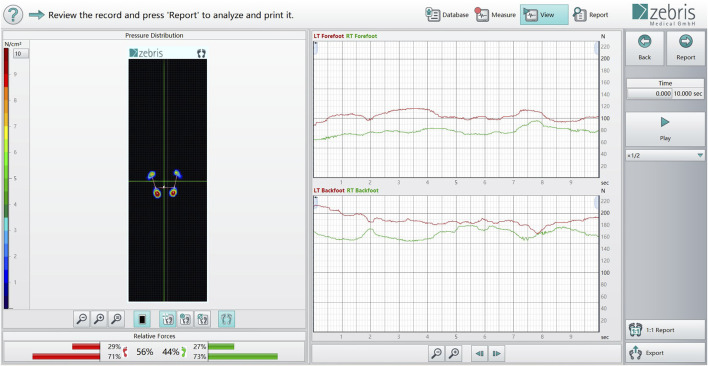
Stabilometric measurement platform and data acquisition software interface used for COP analysis. **(A)** A pressure-sensing platform (Zebris system) was used to record plantar pressure distribution and COP trajectories during static balance assessment. **(B)** Zebris data acquisition software interface displaying real-time COP trajectory and stabilometric signals in the mediolateral and anteroposterior directions. Abbreviations: COP, center of pressure.

Static balance was assessed using a Zebris FDM 2.0 plantar pressure platform (Zebris Medical, Isny im Allgäu, Germany) (Figure 1). This platform measures vertical ground reaction forces through an array of capacitive pressure sensors distributed across a 203 cm × 54 cm sensing surface. The system operates at a sampling frequency of 120 Hz, enabling high-resolution temporal tracking of plantar pressure distribution. All trials consisted of a fixed 20-s standing period. The entire recording interval was used for COP computation. No event triggers, segmentation procedures, or time-window selection algorithms were applied.

COP trajectories were continuously sampled at 120 Hz during each 20-s trial. However, the statistical analyses were not performed on individual time-series samples. Instead, the Zebris software calculated summary COP outcome measures, including sway area, mediolateral displacement (COP-X), and anteroposterior displacement (COP-Y), for each trial. Raw pressure signals and COP trajectories were processed automatically using the manufacturer’s proprietary software. No additional filtering or smoothing procedures were applied by the investigators. The sensing principle relies on pressure-induced changes in capacitance: vertical force applied to the platform deforms sensor cells, altering capacitance values. Corresponding signals are converted into pressure maps and used to compute the COP coordinates at each time frame.

### 2.3 Balance assessment protocol

**FIGURE 2:**
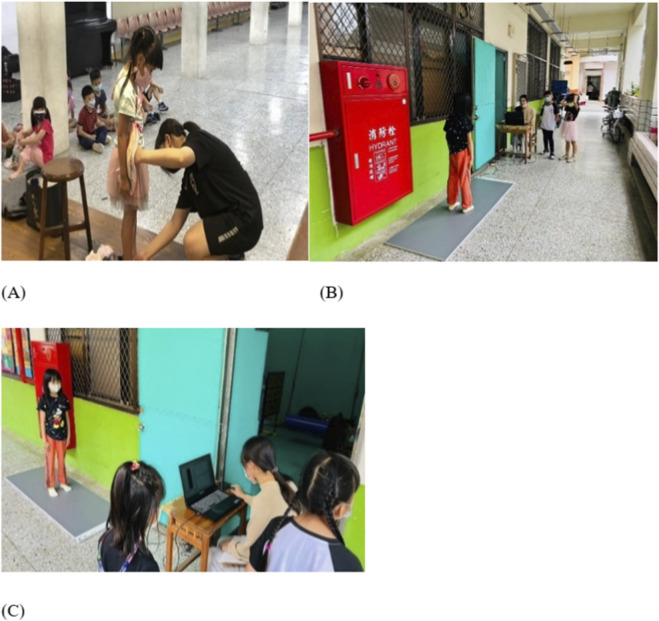
Experimental setup and anthropometric measurement for pediatric balance assessment. **(A)** Static balance measurement obtained using a pressure-sensing platform and simultaneous COP recording. **(B)** Overview of the experimental setup illustrating participant position relative to the pressure platform and data acquisition laptop. **(C)** Anthropometric measurement of leg length used for normalization of COP parameters. Abbreviations: COP, center of pressure.

Static balance was assessed using the pressure-sensing platform under standardized foot placement and posture constraints. Each participant stood barefoot with feet parallel and shoulder-width apart, arms relaxed alongside the trunk, and gaze directed toward a fixed visual target positioned at eye level approximately 2 m ahead (Figure 2).

Each participant completed three valid trials under each visual condition. A trial was considered valid if the participant maintained the required standing posture throughout the 20-s recording period without stepping, excessive body movement, or loss of balance. The mean value obtained from the three valid trials was used for subsequent statistical analyses. Each trial involved a 20-s quiet standing task performed under two sensory conditions: open eyes (OE) and closed eyes (CE). Under the OE condition, participants were instructed to maintain visual fixation on the target to ensure consistent visual input. After a brief rest, the participants performed the trial under the CE condition, maintaining the same posture with visual input removed. The participants were instructed to minimize voluntary body movement, such as stepping, excessive sway, or trunk rotation, to preserve signal integrity. Three consecutive trials were conducted under each condition, with short between-trial rest intervals implemented to minimize fatigue. All measurements were supervised by trained research personnel to ensure protocol compliance and participant safety. Trials were excluded from analysis if participants failed to maintain the required stance or violated task constraints. Data acquisition and monitoring were performed by the same research personnel to maintain procedural consistency.

### 2.4 Data processing

For each 20-s trial, raw plantar pressure data were sampled at 120 Hz. Time-series pressure matrices were exported from the Zebris software for subsequent offline processing. For each participant and condition, the COP outcome measures obtained from the three valid trials were averaged to generate a single representative value for subsequent statistical analyses. Therefore, the unit of analysis was the participant rather than individual COP time-series observations. The COP parameters exported from the Zebris software included sway area, mediolateral displacement (COP-X), and anteroposterior displacement (COP-Y). For each participant, parameter values obtained from the three valid trials were averaged to reduce trial-to-trial variability and provide a representative estimate of postural performance. Data were visually inspected to remove artifacts resulting from excessive movement.

#### 2.4.1 COP calculation

The center-of-pressure (COP) coordinates were calculated using the embedded processing algorithms of the Zebris FDM pressure platform software (Zebris Medical GmbH, Isny, Germany). According to the manufacturer’s technical documentation, COP coordinates are computed as pressure-weighted averages of the sensor coordinates: COP coordinates were computed as the weighted average of pressure sensor forces across all sensing elements:

$${\text{COP}}_{x}=\Sigma \left(F_{i}\;\cdot \;x_{i}\right)/\Sigma F_{i}$$

$${\text{COP}}_{\gamma }=\Sigma \left(F_{i}\;\cdot \;y_{i}\right)/\Sigma F_{i}$$

Expanded form with index notation:

$${\text{COP}}_{x}=\left(\Sigma_{i=1}^{n}|n|\;|F_{i}|\;|x_{i}\right)/\left(\Sigma_{i=1}^{n}\;F_{i}\right)$$

$${\text{COP}}_{\gamma }=\left(\Sigma_{i=1}^{n}|n|\;|F_{i}|\;|y_{i}\right)/\left(\Sigma_{i=1}^{n}\;F_{i}\right)$$

where (x_i) and (y_i) denote the mediolateral (ML) and anteroposterior (AP) coordinates of sensor (i), respectively, and (P_i) represents the pressure recorded by sensor (i). Both coordinates were defined within the horizontal support-surface plane of the pressure platform rather than the vertical direction.

#### 2.4.2 Confidence ellipse area equation (COP sway area)

COP sway area values were exported directly from the Zebris software environment. The COP sway area was defined as the area of the 95% prediction ellipse derived from the covariance structure of COP coordinates. Because COP trajectories represent a two-dimensional distribution, the ellipse scaling factor was determined using the 95th percentile of the chi-square distribution with two degrees of freedom ( $\chi_{0.95,2}^{2}=5.991$ ) rather than the one-dimensional normal critical value of 1.96. Following Schubert and Kirchner (2014), the 95% ellipse area was calculated as:

$$\left[Area_{95}=\pi \times \chi_{0.95,2}^{2}\times \sqrt{\lambda_{1}\lambda_{2}}\right]$$

where $\lambda_{1}$ and $\lambda_{2}$ are the first and second eigenvalues of the COP covariance matrix, respectively, and $\chi_{0.95,2}^{2}=5.991$ . This formulation was used because COP sway area is derived from two-dimensional COP dispersion rather than a one-dimensional confidence interval. The previously used normal-distribution critical value (1.96) is appropriate for one-dimensional confidence intervals but does not correctly represent the dispersion of a two-dimensional COP distribution.

#### 2.4.3 Normalization

To reduce anthropometric scaling bias, COP parameters were normalized using leg length. The choice of leg length was based on its biomechanical relevance to the inverted-pendulum model of quiet standing. Because COP displacement reflects oscillatory motion around the ankle joint, lower-limb length provides a direct estimate of the effective lever arm associated with postural sway. From an inverted-pendulum perspective, COP displacement is influenced not only by neuromotor control but also by body geometry. Compared with body mass, which primarily affects force generation, and standing height, which includes body segments not directly involved in lower-limb sway mechanics, leg length provides a more biomechanically relevant scaling factor for COP-based balance assessment (Winter, 1995; Hof, 1996). To obtain dimensionally consistent normalized COP variables, length-based COP displacement parameters and area-based COP sway parameters were normalized separately according to their physical dimensions. Following biomechanical scaling principles, linear COP displacement variables were normalized by leg length, whereas COP sway area was normalized by squared leg length (Hof, 1996; Chiari et al., 2020).

For linear COP displacement parameters:

$$COP_{X,norm}=\frac{COP_{X,raw}}{LL}$$

$$COP_{Y,norm}=\frac{COP_{Y,raw}}{LL}$$

$$Area_{norm}=\frac{Area_{raw}}{LL^{2}}$$

where (LL) represents leg length (m). Linear displacement variables were normalized by leg length, whereas COP sway area was normalized by squared leg length because sway area represents a two-dimensional measure (Schubert and Kirchner, 2014).

### 2.5 Statistical analysis

Because the analyses were performed on participant-level summary variables rather than raw COP trajectories, each participant contributed one observation per outcome measure. Consequently, temporal autocorrelation among raw COP samples did not affect the statistical analyses. Data quality was examined before statistical analysis. Trials affected by obvious movement artifacts, loss of foot contact, or failure to maintain the required standing posture were excluded and repeated. No participant-level data were excluded after completion of data collection. Therefore, all 90 participants were included in the final analyses. Statistical analyses were performed using SPSS (version 23.0, IBM, Armonk, NY, United States). Descriptive statistics were computed for all COP parameters in both the raw and the normalized datasets. Data are presented as mean ± standard deviation values. Shapiro–Wilk tests were performed to assess normality. Because the study employed balanced group sizes and ANOVA is generally robust to moderate deviations from normality, all primary analyses were conducted using two-way ANOVA.

The relatively balanced sample sizes across groups reduce the sensitivity of ANOVA to minor departures from normality. For variables satisfying normality assumptions, a two-way analysis of variance (ANOVA) was performed using age group (4, 6, and 10 years) and sex (male vs. female) as between-subject factors. When significant age × sex interactions were observed, simple main effects analyses were performed to decompose interaction patterns. If no significant interaction was observed, main effects were interpreted directly.

Post hoc pairwise comparisons were performed using the Bonferroni correction procedure to control for inflated Type I error associated with multiple comparisons. When the assumption of normality was violated, nonparametric Kruskal–Wallis tests were applied. The variables requiring nonparametric analysis and their corresponding results are reported in the Results section. Prior to inferential analyses, normality of the COP variables was evaluated using the Shapiro–Wilk test. The results are presented in Table 2. All variables demonstrated p-values greater than 0.05, indicating no statistically significant deviations from normality. Accordingly, parametric analyses were retained for subsequent statistical testing.

**TABLE 2:** Assessment of normality assumptions for center-of-pressure (COP) variables using the Shapiro–Wilk test.

Variable	Shapiro–Wilk *p*
OE area	0.112
OE-X	0.086
OE-Y	0.218
CE area	0.093
CE-X	0.071
CE-Y	0.147

OE, open-eyes condition; CE, closed-eyes condition; Area = COP, sway area; X, mediolateral COP, displacement; Y, anteroposterior COP, displacement. Shapiro–Wilk tests were conducted to assess the normality of the COP, variables prior to inferential analyses. All variables yielded p-values greater than .05, indicating no statistically significant departures from normality. Therefore, parametric analyses were considered appropriate for the present dataset. Given the balanced design of the study (n = 30 per age group), minor deviations from normality would be unlikely to substantially affect the validity of the ANOVA, results.

Effect sizes were estimated using η^2^ and interpreted as small (η^2^ ≥ 0.01), medium (η^2^ ≥ 0.06), or large (η^2^ ≥ 0.14). Statistical significance was evaluated using an alpha level of 0.05. Exact p-values are reported whenever *p* ≥ 0.001, whereas *p*-values smaller than 0.001 are reported as *p* < 0.001. Effect sizes are presented as partial eta-squared (ηp^2^). For variables violating normality assumptions, a nonparametric Kruskal–Wallis test was performed to detect intergroup differences. Furthermore, CV was used to characterize relative variability within each COP parameter and should not be interpreted as a direct measure of interindividual differences. To characterize the relative variability of COP parameters, the coefficient of variation CV was calculated for each outcome variable. The CV was defined as:

$$\left[CV\left(\%\right)=\frac{SD}{Mean}\times 100\right]$$

where SD represents the standard deviation and Mean represents the arithmetic mean of the corresponding COP parameter. Because the CV expresses variability relative to the magnitude of the measurement, it facilitates comparisons across variables with different units or scales (Atkinson and Nevill, 1998). This dual-track inferential approach enabled investigation of whether anthropometric normalization influenced statistical outcomes and facilitated computational validation of scaling sensitivity in developmental COP data.

## 3 Results

Although age-related effects represented the primary focus of this study, age and sex interactions were also examined to determine whether developmental trajectories differed between boys and girls.

### 3.1 Effects of age and sex on COP parameters

All variables satisfied the normality assumption according to the Shapiro–Wilk test (all p > 0.05). Consequently, no nonparametric analyses were required. Results of the Shapiro–Wilk normality tests are summarized in Table 2. All COP variables satisfied the normality assumption (all *p* > 0.05). Consequently, two-way ANOVA was used as the primary analytical approach. The raw and normalized datasets were analyzed to detect potential scaling bias in developmental inference. Two-way ANOVA revealed significant age × sex interactions in anteroposterior COP (COP-X) displacement but not COP sway area or mediolateral COP (COP-Y) displacement. Significant age × sex interaction effects were observed for COP-X displacement under both visual conditions. Under the OE condition, significant interaction effects were identified for both the raw dataset, *F* (2, 84) = 6.092, *p* = 0.003, η^2^ = 0.127, and the leg-length-normalized dataset, *F* (2, 84) = 6.445, *p* = 0.002, η^2^ = 0.133. Similarly, under the CE condition, significant interaction effects were detected for both the raw dataset, *F* (2, 84) = 4.532, *p* = 0.014, η^2^ = 0.097, and the normalized dataset, *F* (2, 84) = 3.785, *p* = 0.027, η^2^ = 0.083. No significant age × sex interactions were observed for the remaining COP variables. These findings indicate that age- and sex-dependent differences were evident primarily in mediolateral COP displacement rather than in COP sway area or anteroposterior displacement. Detailed descriptive statistics for the raw and normalized datasets are presented in Tables 3, 4, whereas the corresponding ANOVA results are summarized in Table 5.

**TABLE 3:** Descriptive statistics for raw and normalized COP parameters across age and sex groups.

Group variables	4-year-old group M ± SD	6-year-old group M ± SD	10-year-old group M ± SD	Boys M ± SD	Girls M ± SD
OE-COP area (Raw)	939.18 ± 597.45	523.61 ± 323.30	256.41 ± 193.62	495.10 ± 472.10	651.03 ± 504.94
OE-COP area (LL-norm.)	18.71 ± 11.66	8.88 ± 5.52	3.49 ± 2.83	9.00 ± 9.00	11.72 ± 10.55
OE-COP-X (Raw)	17.21 ± 14.32	8.83 ± 7.59	10.90 ± 9.86	11.21 ± 8.85	13.41 ± 13.50
OE-COP-X (LL-norm.)	0.34 ± 0.28	0.14 ± 0.12	0.13 ± 0.14	0.18 ± 0.13	0.23 ± 0.27
OE-COP-Y(Raw)	22.75 ± 9.47	20.36 ± 11.11	24.37 ± 12.70	22.87 ± 10.73	22.18 ± 11.69
OE-COP-Y (LL-norm.)	0.45 ± 0.19	0.34 ± 0.18	0.32 ± 0.15	0.38 ± 0.18	0.36 ± 0.19
CE-COP area (Raw)	995.17 ± 588.71	857.07 ± 680.92	365.40 ± 257.18	665.21 ± 587.53	813.20 ± 608.79
CE-COP area (LL-Norm.)	19.71 ± 11.36	14.61 ± 11.83	4.94 ± 3.60	11.92 ± 11.22	14.26 ± 11.57
CE-COP-X (Raw)	13.85 ± 10.83	10.18 ± 8.53	12.04 ± 8.92	11.3 1 ± 8.73	12.73 ± 10.24
CE-COP-X (LL-norm.)	0.27 ± 0.21	0.17 ± 0.14	0.16 ± 0.11	0.18 ± 0.13	0.22 ± 0.19
CE-COP-Y(Raw)	22.30 ± 12.46	22.29 ± 11.21	25.06 ± 12.35	21.84 ± 10.33	24.58 ± 13.35
CE-COP-Y (LL-norm.)	0.44 ± 0.25	0.38 ± 0.19	0.33 ± 0.15	0.36 ± 0.17	0.40 ± 0.23

OE-COP, area represents the COP, sway area under the OE, condition. OE-COP-X, and OE-COP-Y, represent mediolateral and anteroposterior COP, displacements, respectively, under the OE, condition; CE-COP, area represents the COP, sway area under the CE, condition. CE-COP-X, and CE-COP-Y, represent mediolateral and anteroposterior COP, displacements, respectively, under the CE, condition. Data are presented as mean ± SD, values. Raw = original COP, values without anthropometric scaling. LL-Norm. = COP, values normalized using leg length according to the procedures described in Section 2.4.3. For displacement variables, LL-Norm. Indicates normalization by leg length (LL), whereas COP, sway area was normalized by LL^2^. Abbreviations: CE, closed eyes; COP, center of pressure; OE, open eyes; SD, standard deviation.

**TABLE 4:** Sex-specific descriptive statistics for raw and normalized COP parameters across age groups.

Group variables	4-year-old group		6-year-old group		10-year-old group	
	Boys	Girls	Boys	Girls	Boys	Girls
	M ± SD	M ± SD	M ± SD	M ± SD	M ± SD	M ± SD
OE-COP area (mm^2^) (raw)	877.91 ± 59.398	1,000.44 ± 610.98	426.33 ± 249.52	620.88 ± 366.02	181.04 ± 111.38	331.78 ± 230.39
OE-COP area (mm^2^) (LL-Norm.)	17.21 ± 10.63	20.22 ± 12.80	7.37 ± 4.22	10.39 ± 6.35	2.43 ± 1.60	4.56 ± 3.41
OE-COP-X (mm) (raw)	11.12 ± 7.31	23.30 ± 17.08	8.46 ± 6.20	9.20 ± 8.98	14.06 ± 11.73	7.73 ± 6.52
OE-COP-X (mm) (LL-norm.)	0.22 ± 0.14	0.46 ± 0.34	0.15 ± 0.11	0.15 ± 0.15	0.19 ± 0.15	0.08 ± 0.12
OE-COP-Y (mm) (raw)	23.02 ± 10.77	22.49 ± 8.34	20.01 ± 10.92	20.01 ± 11.66	24.06 ± 10.91	24.06 ± 14.67
OE-COP-Y (mm) (LL-norm.)	0.46 ± 0.21	0.45 ± 0.17	0.37 ± 0.19	0.33 ± 0.19	0.32 ± 0.13	0.32 ± 0.19
CE-COP area (mm^2^) (raw)	928.45 ± 593.13	1,061.87 ± 597.15	766.43 ± 682.31	947.70 ± 690.8	300.76 ± 200.02	430.04 ± 296.72
CE-COP area (mm^2^) (LL-norm.)	18.36 ± 11.31	21.06 ± 11.63	13.37 ± 12.08	15.86 ± 11.87	4.02 ± 2.83	5.87 ± 4.12
CE-COP-X (mm) (raw)	10.36 ± 7.02	17.33 ± 12.95	8.25 ± 7.97	12.11 ± 8.90	15.33 ± 9.93	8.74 ± 6.54
CE-COP-X (mm) (LL-norm.)	0.21 ± 0.14	0.34 ± 0.24	0.14 ± 0.14	0.20 ± 0.15	0.20 ± 0.12	0.11 ± 0.08
CE-COP-Y (mm) (raw)	20.94 ± 11.69	23.66 ± 13.46	20.54 ± 9.84	23.91 ± 12.48	23.94 ± 9.71	26.18 ± 14.80
CE-COP-Y (mm) (LL-norm.)	0.41 ± 0.23	0.47 ± 0.27	0.36 ± 0.17	0.39 ± 0.21	0.31 ± 0.11	0.35 ± 0.19

OE-COP, area represents the COP, sway area under the OE, condition. OE-COP-X, and OE-COP-Y, represent mediolateral and anteroposterior COP, displacements, respectively, under the OE, condition; CE-COP, area represents the COP, sway area under the CE, condition. CE-COP-X, and CE-COP-Y, represent mediolateral and anteroposterior COP, displacements, respectively, under the CE, condition. Data are presented as mean ± SD, values. Raw = original COP, values without anthropometric scaling. LL-Norm. = COP, values normalized using leg length according to the procedures described in Section 2.4.3. For displacement variables, LL-Norm. Indicates normalization by leg length (LL), whereas COP, sway area was normalized by LL^2^. Abbreviations: CE, closed eyes; COP, center of pressure; OE, open eyes; SD, standard deviation.

**TABLE 5:** Two-way ANOVA results for raw and normalized COP parameters across age and sex groups.

Group variables	Age				Sex				Age* sex		
	*F*	*P*	$\eta^{2}$	*Sig*	*F*	*P*	$\eta^{2}$	*Sig*	*F*	*P*	$\eta^{2}$
OE-COP area (Raw)	21.456^**^	<0.001	0.338	4 > 6 (<0.001) 4 > 10 (<0.001) 6 > 10 (0.038)	3.306	0.073	0.038	None	0.060	0.942	0.001
OE-COP area (LL-Norm.)	30.687^**^	<0.001	0.422	4 > 6 (<0.001) 4 > 10 (<0.001)	2.863	0.094	0.033	None	0.033	0.097	0.001
OE-COP-X (Raw)	5.321^**^	0.007	0.112	4 > 6 (0.007)	1.011	0.318	0.012	None	6.092^**^	0.003	0.127
OE-COP-X (LL-norm.)	11.797^**^	<0.001	0.219	4 > 6 (<0.001) 4 > 10 (<0.001)	1.558	0.215	0.018	None	6.445^**^	0.002	0.133
OE-COP-Y(Raw)	0.920	0.402	0.022	None	0.069	0.793	0.001	None	0.001	0.999	0.000
OE-COP-Y (LL-norm.)	4.493^*^	0.014	0.098	4 > 10 (0.017)	0.166	0.685	0.002	None	0.051	0.950	0.001
CE-COP area (Raw)	11.084^**^	<0.001	0.209	4 > 10 (<0.001) 6 > 10 (0.002)	1.662	0.201	0.019	None	0.021	0.979	0.001
CE-COP area (LL-Norm.)	17.592^**^	<0.001	0.295	4 > 10 (<0.001) 6 > 10 (<0.001)	1.292	0.259	0.015	None	0.015	0.985	0.000
CE-COP-X (Raw)	1.204	0.305	0.028	None	0.538	0.465	0.006	None	4.532^*^	0.014	0.097
CE-COP-X (LL-norm.)	4.708^*^	0.012	0.101	4 > 6 (0.047) 4 > 10 (0.018)	1.049	0.309	0.012	None	3.785^*^	0.027	0.083
CE-COP-Y(Raw)	0.523	0.595	0.012	None	1.157	0.285	0.014	None	0.013	0.988	0.000
CE-COP-Y (LL-norm.)	2.218	0.115	0.051	None	1.045	0.310	0.012	None	0.016	0.984	0.000

Two-way ANOVA, was performed to analyze the main effects of age and sex as well as their interaction effects (age × sex) on COP, parameters. Significance: **p < 0.01 and *p < 0.05. *None* indicates nonsignificant effect. OE-COP, area represents the COP, sway area under the OE, condition. OE-COP-X, and OE-COP-Y, represent mediolateral and anteroposterior COP, displacements, respectively, under the OE, condition; CE-COP, area represents the COP, sway area under the CE, condition. CE-COP-X, and CE-COP-Y, represent mediolateral and anteroposterior COP, displacements, respectively, under the CE, condition. Effect sizes are reported as η^2^. Raw = original COP, values without anthropometric scaling. LL-Norm. = COP, values normalized using leg length according to the procedures described in Section 2.4.3. For displacement variables, LL-Norm. Indicates normalization by leg length (LL), whereas COP, sway area was normalized by LL^2^. Abbreviations: ANOVA, analysis of variance; CE, closed eyes; COP, center of pressure; OE, open eyes.

Overall, age-related developmental trends were largely consistent between the raw and normalized datasets. However, leg-length normalization altered the statistical outcomes of several COP variables, suggesting that body-size scaling influenced the sensitivity of age-group comparisons.

For the raw dataset, significant age effects were observed for OE-COP area, OE-COP-X displacement, and CE-COP area. Under the OE condition, COP sway area differed significantly among age groups, *F* (2, 84) = 21.456, *p* < 0.001, η^2^ = 0.338. Post hoc analyses indicated that the 4-year-old group exhibited a larger COP sway area than both the 6-year-old (*p* < 0.001) and 10-year-old groups (*p* < 0.001), and the 6-year-old group exhibited a larger COP sway area than the 10-year-old group (*p* = 0.038). In addition, OE-COP-X displacement demonstrated a significant age effect, *F* (2, 84) = 5.321, *p* = 0.007, η^2^ = 0.112, with the 4-year-old group exhibiting greater displacement than the 6-year-old group (*p* = 0.007).

Under the CE condition, COP sway area also differed significantly across age groups, *F* (2, 84) = 11.084, *p* < 0.001, η^2^ = 0.209, with both the 4-year-old (*p* < 0.001) and 6-year-old groups (*p* = 0.002) exhibiting larger sway areas than the 10-year-old group. No significant age effects were observed for CE-COP-X displacement, *F* (2, 84) = 1.204, *p* = 0.305, η^2^ = 0.028, or CE-COP-Y displacement, *F* (2, 84) = 0.523, *p* = 0.595, η^2^ = 0.012.

Following leg-length normalization, similar developmental patterns were observed, although additional significant age effects emerged. Under the OE condition, normalized COP sway area demonstrated a significant age effect, *F* (2, 84) = 30.687, *p* < 0.001, η^2^ = 0.422, with the 4-year-old group exhibiting larger sway areas than both the 6-year-old (*p* < 0.001) and 10-year-old groups (*p* < 0.001). Normalized OE-COP-X displacement also differed significantly among age groups, *F* (2, 84) = 11.797, *p* < 0.001, η^2^ = 0.219, with the 4-year-old group exhibiting greater displacement than both the 6-year-old (*p* < 0.001) and 10-year-old groups (*p* < 0.001). Furthermore, normalized OE-COP-Y displacement revealed a significant age effect, *F* (2, 84) = 4.493, *p* = 0.014, η^2^ = 0.098, with the 4-year-old group exhibiting greater displacement than the 10-year-old group (*p* = 0.017), a difference that was not detected in the raw dataset.

Under the CE condition, normalized COP sway area remained significantly different among age groups, *F* (2, 84) = 17.592, *p* < 0.001, η^2^ = 0.295, with both the 4-year-old (*p* < 0.001) and 6-year-old groups (*p* < 0.001) exhibiting larger sway areas than the 10-year-old group. In addition, normalized CE-COP-X displacement demonstrated a significant age effect, *F* (2, 84) = 4.708, *p* = 0.012, η^2^ = 0.101, with the 4-year-old group exhibiting greater displacement than both the 6-year-old (*p* = 0.047) and 10-year-old groups (*p* = 0.018). This age-related difference was not observed in the corresponding raw analysis. No significant age effect was detected for normalized CE-COP-Y displacement, F (2, 84) = 2.218, *p* = 0.115, η^2^ = 0.051.

To further quantify the influence of anthropometric normalization, changes in statistical significance and p-value magnitude were compared between raw and normalized COP analyses. Two COP variables changed from non-significant to significant following normalization, whereas no variables changed from significant to non-significant. Furthermore, *p*-values decreased for three variables after normalization (Table 6). These findings indicate that anthropometric normalization generally enhanced statistical sensitivity without masking previously detected effects.

**TABLE 6:** Changes in statistical significance and *p*-values following leg-length normalization.

Variable	Raw *p*	LL-norm. *P*	Direction
OE area	<0.001	<0.001	↓
OE-X	0.007	<0.001	↓
OE-Y	0.402	0.014	NS→Sig
CE area	<0.001	<0.001	↓
CE-X	0.305	0.012	NS→Sig
CE-Y	0.595	0.115	↓

Raw = original COP, values without anthropometric scaling; LL-Norm. = leg-length-normalized COP, values. ↓ indicates a reduction in *p*-value after normalization. NS→Sig indicates a change from non-significant (*p* ≥ .05) to statistically significant (*p* < .05) following normalization. This table is provided to illustrate the extent to which leg-length normalization altered the statistical sensitivity of age-group comparisons. Leg-length normalization resulted in two variables changing from non-significant to significant, no variables changing from significant to non-significant, nine variables exhibiting lower *p*-values, and five variables exhibiting higher *p*-values relative to the raw analyses.

Regarding sex-related differences, overall patterns were consistent between the raw and normalized datasets. No COP parameter differed significantly between boys and girls. The observed changes suggest that leg-length normalization may increase statistical sensitivity within the present dataset. However, because only a single normalization strategy was evaluated, these findings should be interpreted as preliminary evidence rather than definitive proof of superiority over alternative anthropometric scaling approaches.

### 3.2 Interaction effect of age and sex on COP-X displacement

Post hoc analyses of the significant age × sex interactions for COP-X displacement under both visual conditions are summarized in Table 7. To further clarify the interaction effects, age-related comparisons were conducted separately for boys and girls, and sex-related comparisons were performed within each age group. Under the OE condition, the raw and leg-length-normalized datasets demonstrated similar patterns. For boys, no significant age-related differences in COP-X displacement were observed in either the raw dataset, *F* (2, 42) = 1.541, *p* = 0.226, η^2^ = 0.068, or the normalized dataset, *F* (2, 42) = 1.307, *p* = 0.281, η^2^ = 0.059. In contrast, significant age effects were identified among girls in both the raw dataset, *F* (2, 42) = 8.011, *p* = 0.001, η^2^ = 0.276, and the normalized dataset, *F* (2, 42) = 11.919, *p* < 0.001, η^2^ = 0.362. Post hoc analyses revealed that the 4-year-old girls exhibited significantly greater COP-X displacement than both the 6-year-old girls (*p* = 0.002) and the 10-year-old girls (*p* < 0.001) in both analytical tracks.

**TABLE 7:** Main effects of significant age × sex interactions on raw and normalized anteroposterior COP displacement.

Group variables	Boys				Girls				Age			
	*F*	*P*	$\eta^{2}$	*Sig*	*F*	*P*	$\eta^{2}$	*Sig*	Age	*t*	*P*	*Sig*
OE-COP-X (Raw)	1.541	0.226	0.068	None	8.011^**^	0.001	0.276	4 > 6 (0.002) 4 > 10 (<0.001)	4	−2.540	0.017	M > F
									6	−0.262	0.795	None
									10	1.826	0.079	None
OE-COP-X (LL-norm.)	1.307	0.281	0.059	None	11.919^**^	<0.001	0.362	4 > 6 (0.002) 4 > 10 (<0.001)	4	−2.487	0.019	M > F
									6	−0.168	0.867	None
									10	2.024	0.053	None
CE-COP-X (Raw)	2.811	0.071	0.118	6 < 10 (0.026)	2.910	0.066	0.122	4 > 10 (0.021)	4	−1.833	0.077	None
									6	−1.251	0.221	None
									10	2.147	0.041	F > M
CE-COP-X (LL-norm.)	0.987	0.381	0.045	None	6.219^**^	0.004	0.228	4 > 6 (0.038) 4 > 10 (0.001)	4	−1.782	0.086	None
									6	−1.076	0.291	None
									10	2.229	0.034	F > M

Simple main effect analyses were conducted when two-way analysis of variance revealed significant age × sex interactions. Significance: **p < 0.01 *None* indicates nonsignificant difference. OE-COP-X, represents anteroposterior COP, displacement under the OE, condition, and CE-COP-X, represents anteroposterior COP, displacement under the CE, condition. Effect sizes are reported as η^2^. Raw = original COP, values without anthropometric scaling. LL-Norm. = COP, values normalized using leg length according to the procedures described in Section 2.4.3. For displacement variables, LL-Norm. Indicates normalization by leg length (LL), whereas COP, sway area was normalized by LL^2^. Abbreviations: CE, closed eyes; COP, center of pressure; OE, open eyes.

Sex-specific comparisons further indicated that, under the OE condition, 4-year-old boys exhibited significantly greater COP-X displacement than did 4-year-old girls in both the raw dataset (*t* = −2.540, *p* = 0.017) and the normalized dataset (*t* = −2.487, *p* = 0.019). No significant sex-related differences were detected among the 6-year-old group (raw: *t* = −0.262, *p* = 0.795; normalized: *t* = −0.168, *p* = 0.867) or the 10-year-old group (raw: *t* = 1.826, *p* = 0.079; normalized: *t* = 2.024, *p* = 0.053).

Under the CE condition, differences emerged between the raw and normalized datasets. For boys, a significant age effect was observed in the raw dataset, *F* (2, 42) = 2.811, *p* = 0.071, η^2^ = 0.118, with *post hoc* analyses indicating that the 6-year-old group exhibited significantly smaller COP-X displacement than the 10-year-old group (*p* = 0.026). However, this age-related difference was no longer evident after normalization, *F* (2, 42) = 0.987, *p* = 0.381, η^2^ = 0.045. Among girls, the age effect did not reach statistical significance in the raw dataset, *F* (2, 42) = 2.910, *p* = 0.066, η^2^ = 0.122, although *post hoc* testing indicated that the 4-year-old group exhibited greater COP-X displacement than the 10-year-old group (p = 0.021). After normalization, a significant age effect emerged among girls, *F* (2, 42) = 6.219, *p* = 0.004, η^2^ = 0.228, with the 4-year-old group exhibiting significantly greater COP-X displacement than both the 6-year-old (*p* = 0.038) and 10-year-old groups (*p* = 0.001).

Regarding sex-related comparisons under the CE condition, no significant differences were observed among the 4-year-old group (raw: *t* = −1.833, *p* = 0.077; normalized: *t* = −1.782, *p* = 0.086) or the 6-year-old group (raw: *t* = −1.251, *p* = 0.221; normalized: *t* = −1.076, *p* = 0.291). However, 10-year-old girls exhibited significantly greater COP-X displacement than did 10-year-old boys in both the raw dataset (*t* = 2.147, *p* = 0.041) and the normalized dataset (*t* = 2.229, *p* = 0.034).

To further quantify the influence of normalization on statistical outcomes, a sensitivity comparison was performed (Table 6). Two variables changed from non-significant to significant following leg-length normalization, whereas no variables changed from significant to non-significant. In addition, p-values decreased for the majority of COP variables after normalization, indicating a directional shift toward increased statistical sensitivity.

### 3.3 Variability in COP parameters across age and sex groups

CVs for raw and normalized COP parameters across age and sex groups are presented in Table 8. Notably, the CVs for most COP parameters exceeded 50%, indicating substantial interindividual variability in static balance performance among young children. A comparison of the raw and normalized datasets revealed generally similar variability patterns. Anthropometric normalization did not significantly alter the magnitude or overall trends of variability across age or sex groups. Furthermore, the CVs for most COP parameters did not vary significantly across the age groups or between the sex groups.

**TABLE 8:** CVs for raw and normalized COP parameters across age and sex groups.

Group variables	4-year-old group		6-year-old group		10-year-old group	
	Boys	Girls	Boys	Girls	Boys	Girls
	CV%	CV%	CV%	CV%	CV%	CV%
OE-COP area (mm^2^) (raw)	61.07	68.16	58.95	58.52	69.44	61.52
OE-COP area (mm^2^) (LL-Norm.)	63.29	61.77	61.17	57.27	74.82	66.14
OE-COP-X (mm) (raw)	73.29	65.74	97.62	73.36	84.33	83.38
OE-COP-X (mm) (LL-norm.)	73.45	65.74	98.60	72.82	85.94	79.65
OE-COP-Y (mm) (raw)	37.10	46.79	58.27	51.51	60.97	44.18
OE-COP-Y (mm) (LL-norm.)	38.36	46.87	58.89	50.04	57.62	41.05
CE-COP area (mm^2^) (raw)	56.23	63.88	72.89	89.02	68.99	66.50
CE-COP area (mm^2^) (LL-Norm.)	55.23	61.64	74.83	90.33	70.26	70.46
CE-COP-X (mm) (raw)	74.70	67.83	73.52	96.58	74.81	64.77
CE-COP-X (mm) (LL-norm.)	73.05	68.48	74.54	96.54	74.51	61.31
CE-COP-Y (mm) (raw)	56.88	55.80	52.17	47.22	56.55	40.57
CE-COP-Y (mm) (LL-norm.)	57.84	55.36	53.48	46.27	55.66	35.76

CV% values represent percent CVs, used to quantify interindividual variability in stabilometric parameters; OE-COP, area represents the COP, sway area under the OE, condition. OE-COP-X, and OE-COP-Y, represent mediolateral and anteroposterior COP, displacements, respectively, under the OE, condition; CE-COP, area represents the COP, sway area under the CE, condition. CE-COP-X, and CE-COP-Y, represent mediolateral and anteroposterior COP, displacements, respectively, under the CE, condition. Raw = original COP, values without anthropometric scaling. LL-Norm. = COP, values normalized using leg length according to the procedures described in Section 2.4.3. For displacement variables, LL-Norm. Indicates normalization by leg length (LL), whereas COP, sway area was normalized by LL^2^. Abbreviations: CE, closed eyes; COP, center of pressure; CV, coefficient of variation; OE, open eyes.

Several parameters exhibited relatively high variability. Notably, 6-year-old boys exhibited the largest variability in COP-Y displacement under the OE condition, whereas 6-year-old girls exhibited the largest variability in COP-Y displacement under the CE condition. These findings suggest that postural control during early childhood exhibits substantial variability, with the magnitude of sway varying across sensory conditions and developmental stages.

## 4 Discussion

### 4.1 Effects of anthropometric normalization on COP parameters

We investigated whether anthropometric normalization influences the interpretation of pediatric balance parameters derived from COP measurements. Leg-length normalization produced measurable changes in statistical outcomes across several COP variables. Although the magnitude of these changes varied among parameters, the results indicate that normalization may influence the interpretation of age-related balance differences. Importantly, normalization did not produce random directional changes across outcomes. Instead, p-values decreased in the majority of variables, and no variable changed from significant to non-significant, suggesting a systematic rather than purely stochastic pattern. Specifically, COP sway area, COP-Y displacement, and COP-X displacement exhibited marked differences in both statistical significance and developmental trends between the raw and normalized datasets. These findings suggest that anthropometric scaling improves comparability across age groups by reducing body size–related measurement bias. The findings highlight the importance of normalization in the comparison of balance performance across individuals with different body dimensions.

Maiorov et al. (2025) reported that normalization facilitates the integration and interpretation of postural stability data across heterogeneous populations. Nonetheless, in consideration of the presence of developmental variability, the researchers emphasized a need for caution when interpreting normalized balance metrics in young children. Similarly Hof (1996), demonstrated that biomechanical scaling based on anthropometric parameters effectively reduces size-dependent variance in human movement data. In pediatric populations, developmental differences in body proportions and neuromotor maturation may amplify the effects of anthropometric factors on balance parameters, rendering normalization particularly relevant in childhood-focused studies. Importantly, the observed changes were not uniformly distributed around the significance threshold. No variables changed from significant to non-significant after normalization, whereas two variables changed in the opposite direction. This asymmetry suggests that the observed effects are unlikely to be attributable solely to random statistical fluctuation.

The choice of leg length as the normalization variable was based on biomechanical considerations. During quiet standing, postural sway can be approximated as an inverted-pendulum system in which the lower-limb segment length influences the mechanical characteristics of body oscillation. Leg length therefore provides a direct estimate of the effective lever arm associated with COP displacement. Although alternative anthropometric variables such as standing height, body mass, or estimated center-of-mass height may also be considered for scaling purposes, these variables incorporate additional anatomical or physiological factors that are less directly related to the kinematics of postural sway. These findings demonstrate that anthropometric normalization can enhance the sensitivity of developmental balance assessment by reducing body-size-related bias.

Despite their advantages, explicit normalization strategies have been rarely implemented in studies on pediatric balance. Julienne et al. (2024) reported that many current stabilometric investigations lack standardized references for pediatric populations, which complicates cross-study comparisons. This methodological limitation underscores the relevance of the present study, which directly compared raw and normalized datasets within the same experimental framework. We introduced a dual-track analytical approach in which identical COP datasets are processed in parallel through raw and anthropometrically normalized computational pipelines. Importantly, the innovation of this framework does not reside in the scaling equation itself, because anthropometric normalization has long been recognized in biomechanics (Hof, 1996). Rather, its novelty lies in treating normalization as a computational validation component within the measurement architecture. By simultaneously examining raw and normalized outputs, the framework enables explicit identification of scaling-sensitive parameters and provides a mechanism for evaluating whether developmental conclusions remain stable after correction for body-size effects.

This approach enables direct investigation of how anthropometric normalization influences statistical inference. Our results indicate that certain parameters are more sensitive to normalization, whereas other parameters exhibit relatively stable patterns regardless of scaling. Specifically, anthropometric normalization enhanced the inferential sensitivity of COP sway area and COP-X displacement, allowing clearer detection of age-related differences. By contrast, COP-Y displacement exhibited relatively stable statistical behavior, reflecting lower susceptibility to geometric scaling effects. These findings suggest that normalization requirements vary across stabilometric parameters.

From an engineering and measurement perspective, our dual-track framework represents a refined strategy for evaluating pediatric balance data. Rather than treating normalization as a purely *post hoc* adjustment, the present study conceptualizes it as a methodological component of the signal-processing pipeline. This approach may facilitate the development of standardized data-processing protocols for pediatric stabilometry and improve the reliability of cross-sectional and longitudinal comparisons in childhood balance research. Nevertheless, the use of leg length as the sole scaling variable represents a limitation of the present study. Anthropometric normalization based on leg length does not fully account for interindividual differences in body mass distribution, segment proportions, or center-of-mass location. Future studies should compare alternative scaling models incorporating body mass, stature, or estimated center-of-mass height to determine the optimal normalization strategy for pediatric stabilometry.

It should be acknowledged that the present framework was evaluated using only one anthropometric scaling variable. Although leg length was selected based on biomechanical considerations, alternative normalization approaches using body height, body mass, or estimated center-of-mass height may also be informative. Leg-length normalization enhanced sensitivity within the present dataset. Whether similar improvements would be observed using alternative anthropometric scaling variables remains to be determined. Consequently, the proposed dual-track framework should be interpreted as a proof-of-concept implementation rather than a definitive normalization standard. Future studies should extend the framework into a multi-track architecture to compare multiple anthropometric scaling models and determine whether sensitivity improvements are consistent across normalization strategies. A further limitation is that only one anthropometric scaling variable was evaluated. Although leg length was selected based on biomechanical considerations, the present study did not compare alternative normalization variables such as stature, body mass, or estimated center-of-mass height. Consequently, the observed improvements in statistical sensitivity should be interpreted as evidence supporting leg-length normalization rather than anthropometric normalization in general.

The influence of normalization was further evaluated through a sensitivity comparison (Table 6). Importantly, the observed changes were not randomly distributed around the significance threshold. Two variables changed from non-significant to significant following normalization, whereas no variables changed in the opposite direction. Furthermore, most COP variables exhibited lower p-values after normalization. These findings suggest a systematic influence of leg-length normalization on statistical sensitivity within the present dataset. Nevertheless, the observed changes were derived from comparisons between raw and leg-length-normalized analyses only. Therefore, the present results should be interpreted as evidence specific to the normalization strategy employed rather than as proof of superiority for anthropometric normalization in general.

It should be acknowledged that the observed improvements were identified through comparisons between raw and leg-length-normalized analyses only. Consequently, the present findings cannot determine whether similar sensitivity gains would be observed using alternative anthropometric normalization variables such as stature, body mass, or estimated center-of-mass height. The proposed framework should therefore be interpreted as a proof-of-concept demonstration of leg-length normalization rather than a universal normalization strategy. Because only one normalization variable was examined, the extent to which the observed changes reflect general anthropometric normalization effects rather than leg-length-specific effects remains uncertain.

### 4.2 Developmental changes in pediatric postural control

Age-related variations in COP parameters were observed under both OE and CE conditions. Analysis of the raw dataset indicated that several stabilometric parameters varied significantly across the age groups: COP sway area, COP-X displacement, and COP-Y displacement. However, anthropometric normalization altered the statistical patterns, suggesting that body-size scaling can influence the detection of developmental effects in pediatric balance assessments. The observed developmental trend reflects gradual enhancement of postural stability as neuromotor control and sensory integration mature during childhood. Postural stability increases with age, with younger children typically exhibiting greater postural sway than do older children, which reflects the maturation of postural control mechanisms (Pischetola et al., 2025). The age-related improvements observed in the present study are consistent with the findings of developmental studies demonstrating that postural stability progressively increases during childhood as sensory integration and neuromuscular coordination mature (Assaiante et al., 2005; Schmit et al., 2005). Our findings support this developmental pattern: the 10-year-old group exhibited significantly smaller normalized COP sway area, COP-Y displacement, and COP-X displacement than did the younger groups.

Several age-related differences were detected only after normalization, indicating that anthropometric scaling increases the sensitivity of statistical analyses in the evaluation of developmental balance characteristics. Because body dimensions rapidly change during childhood, raw COP metrics may partially reflect body-size effects rather than purely reflecting neuromotor control. Therefore, normalization helps isolate functional balance performance from morphological influences (Hof, 1996; Winter, 1995). In the present study, the 4-year-old group exhibited a significantly greater COP-Y displacement under the CE condition than did the older groups. This finding suggests that younger children rely more heavily on visual feedback for postural stability and that the removal of visual input reduces stability. Neuroimaging data suggest that postural control relies on distributed neural networks, including the cerebellum and cortical multisensory integration regions, where visual input plays a key role in modulating balance-related neural activity (Dijkstra et al., 2020). Therefore, the greater COP-Y displacement observed in younger children during visual deprivation likely reflects immature sensory integration mechanisms.

COP-X displacement exhibited increased sensitivity to developmental changes after normalization. Stabilometric evaluation can be performed using global sway metrics, such as COP area, as indicators of postural stability. However, directional COP analysis can provide more detailed insights into the strategies underlying postural control. By distinguishing mediolateral and anteroposterior components, the present study offers a nuanced characterization of pediatric balance development. Overall, analyses based on anthropometric normalization detect developmental differences in balance parameters more effectively than do those based on raw data. These findings may assist with methodological refinement in pediatric stabilometry and provide empirical evidence for the incorporation of normalization in evaluation of balance development during childhood. Furthermore, the findings may inform the development of age-appropriate balance assessment tools and support accurate interpretation of pediatric postural control measurements. Several age-related effects were accompanied by moderate-to-large effect sizes, suggesting that the observed differences were not only statistically significant but also practically meaningful. Therefore, the present findings should not be interpreted as evidence that all anthropometric normalization approaches are equivalent. Rather, they specifically support the utility of leg-length normalization within the proposed analytical framework.

Importantly, the interpretation of COP sway magnitude requires caution. While larger sway amplitudes are frequently associated with reduced postural stability, smaller sway values do not necessarily indicate optimal balance control. Contemporary motor-control theories suggest that healthy movement systems exhibit an appropriate degree of variability that supports adaptability and responsiveness to environmental perturbations (Stergiou et al., 2006; Stergiou and Decker, 2011).

### 4.3 Sex-related differences and age–sex interactions

We noted no significant sex-related differences in any COP parameter, regardless of normalization status. This finding suggests that between the ages of 4 and 10 years, boys and girls demonstrate comparable performance in fundamental postural stability tasks during quiet standing. The absence of significant sex-related differences indicates that the maturation of basic postural control mechanisms during early childhood is primarily driven by developmental progression rather than by sex-specific factors. This finding aligns with that of research suggesting that improvements in balance performance during childhood are largely attributable to age-related neuromotor development and accumulated motor experience. In their review of balance training in children with autism spectrum disorders, Baladaniya et al. (2025) reported that progress in postural control is generally associated with developmental maturation and practice exposure rather than sex-related biological differences. Similarly, in their developmental study, Peterson et al. (2006) demonstrated that sensory integration for postural control gradually matures during childhood, enabling children to approach adult-like balance strategies during late childhood.

At this stage, sex-related differences in basic static balance performance are often minimal or absent. This finding is consistent with that of Fujiwara et al. (2011), who investigated age- and sex-related variations in children’s postural control adaptability during periodic floor oscillation under the CE condition. The researchers found that although postural control improved with age, sex-related differences were minimal or absent for most pediatric balance indicators. A possible explanation for the similarity between our study and that of Fujiwara et al. (2011) is that the neuromuscular and sensory integration systems responsible for maintaining upright posture are maturing during early childhood. At this stage, balance control strategies are primarily based on basic sensory feedback mechanisms, such as proprioceptive, vestibular, and visual inputs, rather than sex-specific neuromotor characteristics. Thus, static postural control during quiet standing exhibits limited sex-related differences.

Nevertheless, some studies have reported sex-related differences in balance performance, particularly under challenging sensory conditions. Although the present study revealed no significant sex-related differences in static balance performance, a previous study indicated that school-aged girls exhibit better postural stability—that is, smaller COP sway parameters—than do school-aged boys during quiet standing (Smith et al., 2012). Smaller COP sway is often interpreted as indicating reduced postural instability. However, postural control should not be viewed solely as the minimization of body sway. Effective balance regulation requires continuous sensorimotor adjustments, and excessively reduced sway may reflect a rigid control strategy with diminished adaptability. Therefore, COP sway magnitude should be interpreted within the broader context of postural-control organization rather than as a direct indicator of balance quality. The between-study discrepancy may be attributable to the nature of the experimental task. The balance assessment protocol used in our study involved a basic quiet standing posture with relatively low sensory and motor demands. Such tasks primarily evaluate fundamental postural stability and may not impose sufficient demands to detect subtle sex-related differences in postural control strategies. More demanding balance tasks, such as those involving dynamic perturbations, unstable surfaces, and complex sensory manipulation, may be more sensitive to potential sex-related differences in neuromotor control.

Overall, the present study indicates that during early and middle childhood, fundamental static balance performance is largely similar between boys and girls when assessed under basic standing conditions. Therefore, task complexity and sensory load should be considered in interpreting potential sex-related differences in pediatric balance. Furthermore, no clear correspondence was observed between variability patterns and the group differences identified in the mean COP outcomes.

### 4.4 Variability in pediatric COP parameters

Pediatric balance assessment typically emphasizes mean differences in postural stability parameters. However, very few studies have examined variability in stabilometric measures. These findings indicate substantial relative variability in balance performance across the study population. For most COP parameters, the CVs exceeded 50% across the age groups, regardless of normalization status. The coefficient of variation was used as a dimensionless indicator of relative variability rather than as a direct measure of interindividual differences. This finding indicates substantial variability in postural control strategies across children and implicates interindividual differences in neuromotor development in determining stabilometric performance during childhood. The high variability observed in this study aligns with the concept that postural control undergoes gradual maturation as the nervous system refines sensory integration and motor coordination mechanisms (Assaiante et al., 2005; Schmit et al., 2005). During this developmental stage, children may adopt heterogeneous strategies for maintaining postural stability, which lead to increased variability in COP parameters compared with that noted in adult populations (Winter, 1995).

Our findings showed that variability patterns did not closely mirror the age- and sex-related differences observed in the mean COP measures. This observation indicates that variability-based metrics may provide information that differs from that obtained through traditional mean-based sway measures. A study on variability in human movement reported that variability should not be interpreted merely as measurement noise; rather, it represents an inherent characteristic of the motor control system that reflects adaptive and exploratory processes during movement regulation (Stergiou and Decker, 2011). From this perspective, the high variability observed across groups may reflect natural fluctuations in postural control strategies during childhood. However, the present analyses were not designed to determine whether variability is independently influenced by age or sex. However, the present analyses were not specifically designed to determine whether variability is independently influenced by age or sex. Although coefficients of variation were reported to characterize variability patterns, no dedicated analysis of participant-specific variance structure was performed. Future studies should investigate individual-level variability using complementary approaches such as intraclass correlation analysis. It should be noted that the present study focused primarily on group comparisons and descriptive variability analyses. Regression-based approaches were not performed; therefore, the independent contributions of age, sex, or anthropometric factors to stabilometric variability could not be determined. Future studies may further investigate these relationships using predictive statistical models.

From a methodological perspective, incorporating variability metrics such as the CV provides a complementary dimension to traditional mean-based analyses. Variability in movement is a key characteristic of the motor control system, reflecting adaptive and exploratory processes during motor development (Newell and Corcos, 1993; Stergiou and Decker, 2011). In pediatric balance assessment, increased variability may therefore reflect ongoing sensorimotor learning rather than deficient postural control. It should be noted that the present study focused primarily on group comparisons and descriptive variability analyses. Regression-based approaches were not performed; therefore, the independent contributions of age, sex, or anthropometric factors to stabilometric variability could not be determined. Future studies may further investigate these relationships using predictive statistical models.

These findings suggest that anthropometric normalization may enhance the sensitivity of pediatric balance assessment by reducing body-size-related influences on COP-derived measures. However, because only leg-length normalization was evaluated in the present study, future research should examine alternative scaling strategies and validate these findings in larger and more diverse pediatric populations. The findings provide a descriptive characterization of COP-based balance performance in healthy children aged 4–10 years under both raw and normalized analytical conditions. The present findings may contribute to the development of future assessment frameworks for pediatric balance evaluation. However, the applicability of the proposed approach to atypical motor populations remains to be established. It should be noted that the coefficient of variation reflects relative dispersion within a population and should not be interpreted as a direct measure of interindividual differences. Future studies may benefit from incorporating reliability-oriented indices, such as intraclass correlation coefficients, to further characterize subject-specific variability across repeated measurements. Besides this, because only typically developing children were included, the present findings cannot be generalized to children with atypical motor development, neurological disorders, or developmental impairments. Future studies should investigate whether the observed normalization effects are also present in clinical populations.

The proposed framework provides a methodological approach for evaluating balance performance in typically developing children. Further validation in clinical populations is required before any diagnostic or screening applications can be considered. Because COP processing was performed using the proprietary Zebris software environment, the specific filtering and smoothing algorithms implemented internally by the manufacturer could not be independently verified.

Although leg-length normalization provided additional insights in the present study, raw COP measures remain valuable in several contexts. For example, when evaluating longitudinal changes within the same individual, when anthropometric variability is limited, or when absolute COP values are clinically meaningful, raw measures may offer more direct interpretation. Therefore, the proposed dual-track framework should be viewed as a complementary rather than replacement approach. It should be emphasized that normalization is not universally required for all balance assessments. Raw COP measures may remain appropriate when repeated measurements are obtained from the same individual, when anthropometric variability is minimal, or when clinically interpretable absolute values are of primary interest. In contrast, normalization may be particularly useful when comparisons involve individuals with substantially different body dimensions, such as pediatric populations undergoing rapid growth-related changes.

## 5 Conclusion

We explored age- and sex-related differences in static balance performance among children aged 4, 6, and 10 years by analyzing COP data derived from plantar pressure measurements. By comparing raw and normalized datasets, we comprehensively assessed developmental variation in pediatric postural control. The results revealed that age plays a key role in the development of static balance. Specifically, under the OE condition, the 10-year-old group had a significantly lower COP sway area and directional COP displacement than did the younger groups, indicating progressive maturation of neuromuscular coordination and sensory integration mechanisms. Several balance parameters, including COP-Y displacement under the CE condition, exhibited clearer age-related differences after anthropometric normalization, suggesting that normalization enhances the sensitivity and interpretability of stabilometric analyses in pediatric populations. Leg-length normalization was associated with increased sensitivity for detecting age-related differences in several COP measures within the present cohort.

Substantial interindividual variability was observed in balance performance, with CVs exceeding 50% for most parameters. This high variability likely reflects ongoing development of sensorimotor control during childhood. Although we did not observe consistent sex-related differences in overall balance performance, certain age-specific patterns suggested that postural control strategies vary with developmental stage and sensory conditions. A key contribution of this study is the establishment of a dual-track computational validation framework that integrates raw and normalized COP analyses within a unified signal-processing architecture. Unlike conventional normalization procedures that function solely as preprocessing steps, the proposed framework uses normalization as a validation mechanism to evaluate scaling sensitivity and inferential robustness in pediatric balance assessment. This dual-track analytical framework provides a nuanced understanding of pediatric balance characteristics and highlights the potential role of directional COP metrics in identifying developmental changes in postural control.

Although leg-length normalization revealed additional age-related patterns in the present study, raw COP measures remain valuable in situations where anthropometric variability is limited or where longitudinal within-subject comparisons are the primary objective. Therefore, raw and normalized analyses should be viewed as complementary sources of information rather than competing alternatives. The dual-track framework proposed here is intended to facilitate a more comprehensive interpretation of pediatric balance performance rather than replace conventional raw COP analysis.

Overall, our findings may help refine stabilometric assessment in children, and they suggest that anthropometric normalization should be integrated into evaluations of developmental differences in balance performance. Leg-length normalization may represent a useful approach for reducing body-size-related influences in pediatric balance assessment. Future studies should compare multiple anthropometric scaling variables to determine whether the present findings generalize beyond leg-length normalization. Inaddition to this, future studies should integrate neurophysiological measurements and advanced motion analysis techniques to develop age- and sex-sensitive balance assessment and training protocols, thereby improving early evaluation and targeted intervention for motor development in children. The proposed framework should therefore be interpreted as a methodological validation approach rather than a definitive normalization standard for pediatric stabilometry. Future research should investigate whether the observed improvements in statistical sensitivity are specific to leg-length normalization or generalizable across alternative anthropometric scaling variables.

## Data Availability

The raw data supporting the conclusions of this article will be made available by the authors, without undue reservation.
